# Users of reimbursed glaucoma medications in Finland in 1986–2023: A nationwide study

**DOI:** 10.1111/aos.16803

**Published:** 2024-12-09

**Authors:** E. Lehtonen, A. Tuulonen, S. Leinonen, K. Vepsäläinen, H. Uusitalo‐Jarvinen

**Affiliations:** ^1^ Faculty of Medicine and Health Technology Tampere University Tampere Finland; ^2^ Tays Eye Centre Tampere University Hospital Tampere Finland

**Keywords:** glaucoma, intraocular pressure lowering medications, long‐term trends, medication users

## Abstract

**Purpose:**

The study evaluates the long‐term trends in the number of patients using reimbursed glaucoma medications in Finland.

**Methods:**

Reimbursement data of 193 082 new glaucoma patients in 1986–2023 were collected from the registry of Finnish Insurance Institution (Kela). The numbers of new, current and deceased users of reimbursed glaucoma medications in Finland at end of each year were analysed by gender and in different age groups.

**Results:**

The number of glaucoma reimbursees per total Finnish population increased 2.5‐fold during 37 years. However, in 2015–2023 the data demonstrated an unexpected decelerating trend in the difference between the number of new and deceased users of glaucoma medications (i.e. ‘net change’ in the medication users). In addition, differences in glaucoma medication users between Finland, other Nordic countries and Scotland were detected.

**Conclusions:**

The 37‐year period demonstrates an overall increasing trend in the number of glaucoma medication reimbursees in Finland. However, the slowing growth in the net change during the recent years is to the contrary to what has generally been forecasted in Finland and globally. Future studies, including an updated prediction model, will be required to clarify whether the number of glaucoma patients under treatment will increase, or decrease in Finland in future as well as to figure out the reasons for the variations.

## 1 INTRODUCTION

Glaucoma is a group of eye diseases characterized by optic nerve neuropathy and gradually progressive visual field defects. Globally, glaucoma has been predicted to increase affecting over 100 million people by 2040 and being among leading causes of irreversible blindness (Adelson et al., 2021; Tham et al., 2014).

Historically, elevated intraocular pressure (IOP) has been overemphasized in the glaucoma diagnostics which has affected prevalence rates in epidemiological studies (Kapetanakis et al., 2016). Due to the changing diagnostic paradigms and the challenges of defining early glaucomatous damage in non‐symptomatic glaucoma patients, both over‐ and underdiagnoses of the disease are likely and present (Founti et al., 2018; Heijl et al., 2013; Holm et al., 2022; Johansson et al., 2024). It is estimated that 90% of glaucoma cases remain undiagnosed in developing countries (Bastawrous & Hennig, 2012; Vijaya et al., 2008) and around 50% in developed countries (Johansson et al., 2024). Appropriate case detection and effective treatment represent the key factors for slowing down or halting the progression of glaucoma. IOP lowering eye drops constitute the prevalent treatment option for glaucoma followed by laser and surgical procedures (Leinonen et al., 2024; Sulonen et al., 2024).

The prevalence of primary‐open angle glaucoma has been estimated to be 2%–3% globally with significant age, geographic and ethnic variations (Kapetanakis et al., 2016; Tham et al., 2014). Multiple studies have reported an increasing prevalence of glaucoma which has generally been related to the aging population (Bro et al., 2021; Kolko et al., 2015; Tham et al., 2014; Wang et al., 2019). The number of glaucoma patients has been forecasted to grow globally with a significant part of the growth outside Europe (Tham et al., 2014).

Previous studies from Nordic countries have evaluated databases of pharmacy claims. They have reported nationwide prevalences and incidences of patients using glaucoma medications (Kolko et al., 2015; Slettedal et al., 2020) as well as forecasts and comparisons between Nordic countries (Bro et al., 2021; Goldschmidt et al., 1989). For example, some studies have reported the highest prevalence of glaucoma drug consumption in Norway and Finland and the lowest in Denmark (Bro et al., 2021; Goldschmidt et al., 1989; Ringvold, 1996). Other studies have also estimated that up to half of patients using medications may not have manifest glaucoma (Holm et al., 2022; Vaahtoranta‐Lehtonen et al., 2007). Nevertheless, the number of patients entitled using reimbursed glaucoma medications reflects the burden of glaucoma for the patients themselves as well as for the eye care system.

The purpose of this study is to report and evaluate the changes in the number of patients using reimbursed glaucoma medications (reimbursees) in Finland during 37‐years, presenting to the best of our knowledge the longest follow‐up so far, and to compare the results to Nordic and other countries.

## 2 METHODS

The primarily tax‐financed healthcare system in Finland is based on public services. The Constitution of Finland decrees the public authorities to guarantee adequate social and health care services for everyone residing in the country. Private healthcare services are also available for which the state‐owned Social Insurance Institution (Kela) reimburses a minor proportion of the visits and procedures. In addition, the employers provide occupational health care.

All people with a domicile in Finland and fulfilling the pre‐determined criteria for using glaucoma medications receive full reimbursement (100%) for purchased glaucoma drugs. Upon new glaucoma diagnosis, the public eye care unit or private ophthalmologist fills in ‘Medical Certificate B', which serves as the claim application. Either the patient, or the medical personnel send the application to Kela. Recently, the system automatically sends the application via a digital link. After Kela has granted the reimbursement, patients pay only a surcharge of 4.5 € per pharmacy dispense for each 3‐month period. Kela grants and pays the reimbursements to the pharmacies, maintains the registry of the new, current and deceased reimbursees. The reimbursement data is available since 1986 and captures the whole Finnish population (5.6 million inhabitants in 2023).

**FIGURE 1:**
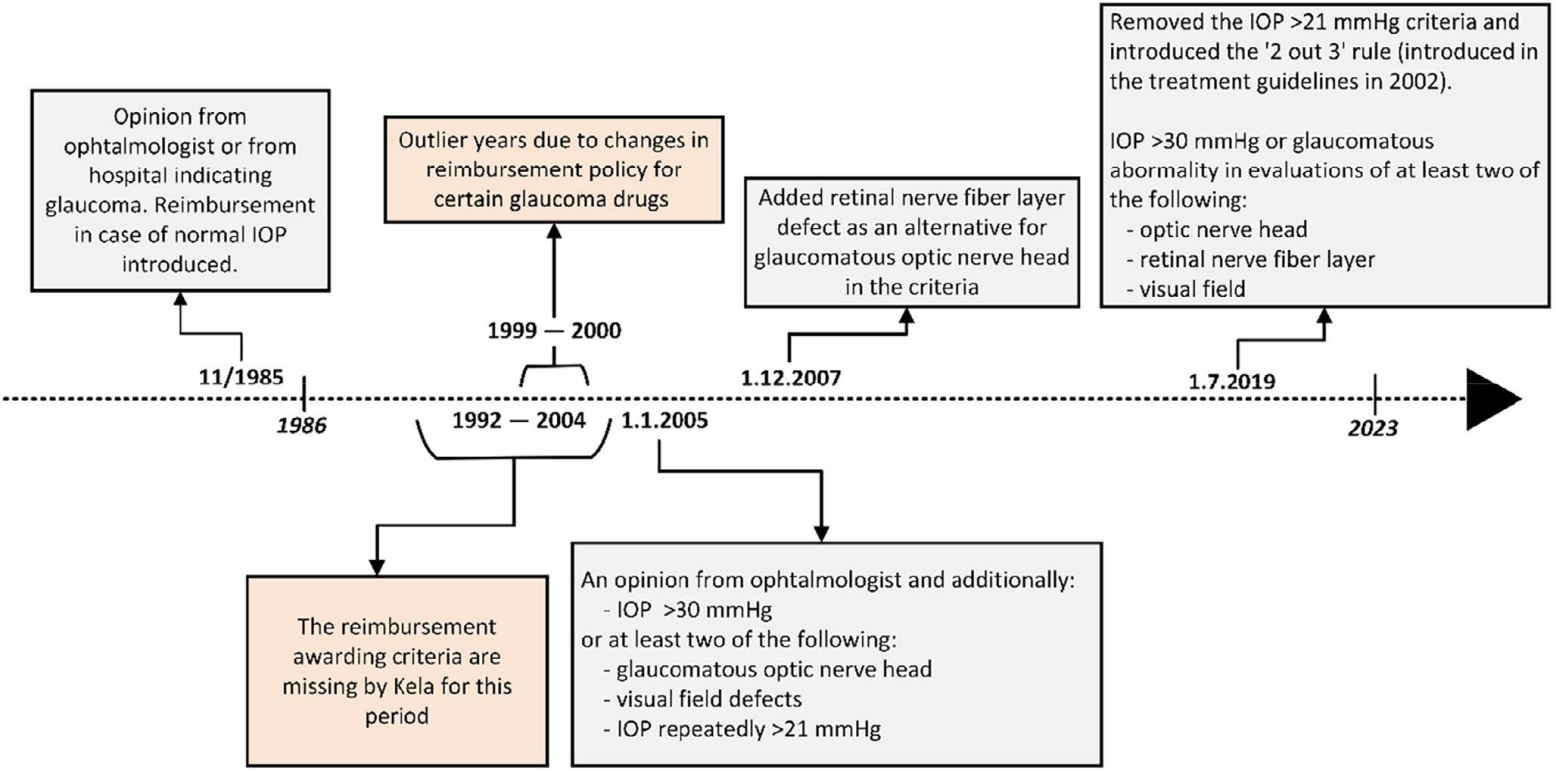
Timeline of glaucoma drug reimbursement policy and awarding criteria changes in Finland by Kela.

The Kela criteria for reimbursing glaucoma medications have changed over the period of 1986–2023 (Figure 1). In summary, an ophthalmologist's clinical evaluation and diagnosis has always formed the basis of the reimbursement claim. In 1985, for the first time, the reimbursement criteria included also normal IOP. The Finnish Current Care Guideline for glaucoma has recommended using ‘2 out of 3’‐rule for diagnosis since 2002, that is at least two out of three findings in the optic nerve head, retinal nerve fibre layer and visual field being glaucomatous, unless the IOP is repeatedly ≥30 mmHg (Tuulonen et al., 2003). Kela criteria has emphasized the requirement of structural and functional abnormalities for diagnosis since 1985.

The four editions of the Finnish Current Care Guideline for glaucoma in 2002–24 have been developed under the leadership of Finnish Medical Society Duodecim in working groups consisting of the members from the Finnish Ophthalmological Society and the Finnish Glaucoma Society (English translations by Tuulonen et al., 2003, 2014; Leinonen et al., 2024). The publicly funded Finnish Current Care Guidelines in all specialties (Finnish Medical Society Duodecim, n.d.) form the basis for national access to care criteria decreed by the Ministry of Social Affairs and Health (Ministry of Social Affairs and Health, 2019).

For this study, the reimbursement data were downloaded from Kela's Info Tray (Kansaneläkelaitos, n.d.). The Info Tray is a publicly available database including nationwide and regional data stratified by gender and 5‐year age groups. The indemnity number 114 for glaucoma identifies all reimbursees of glaucoma medications in 1986–2023. The corresponding Finnish population data were downloaded from the publicly available website of Statistics Finland (Tilastokeskus, n.d.). We also requested the number of rejected reimbursement applications from Kela which has recorded such data since 1992. Additionally, for Sweden we compiled the numbers of individuals with glaucoma prescriptions from the Pharmaceuticals Statistical Database (Socialstyrelsen, n.d.) and similarly for Norway from the Norwegian Prescription Database (Folkehelseinstituttet, n.d.).

At the end of each year, we report (1) the total number of patients entitled to reimbursed glaucoma medications (reimbursees) in different age groups and adjusted to corresponding population (per. 1000 inhabitants) in Finland. Similarly, we also report (2) the number of new reimbursees and (3) the yearly net increase of new reimbursees that is the difference between the number of new and deceased reimbursees. Mortality rate of reimbursees was calculated by dividing the annual number of deceased reimbursees by all persons who had reimbursement rights during the year. R‐statistical software (version 4.3.2) was applied for statistical analysis.

Retrospective register‐based studies do not require ethical approval in Finland and the study material is freely available online apart from the number of rejected reimbursement applications.

## 3 RESULTS

**FIGURE 2:**
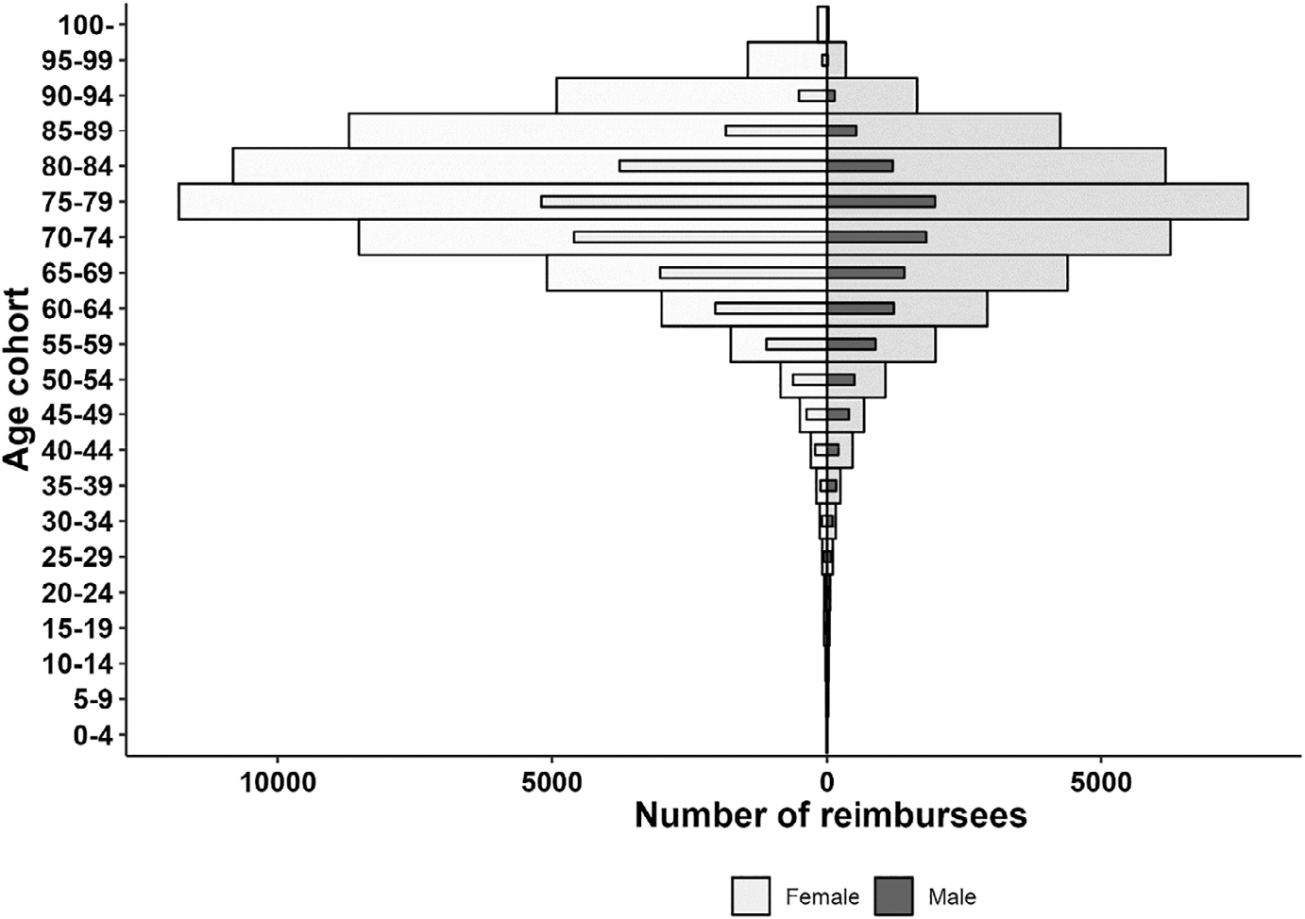
Change in glaucoma medication reimbursee age and gender distribution between 1986 (small darker bars) and 2023 (large bars). Kela didn't report number of over 100‐year‐olds before 2018, so their share is missing from 1986 bars.

Between 1986 and 2023, a total of 193 082 new patients were granted entitlement for using reimbursed glaucoma medications in Finland. The comparison of the age and gender distributions of the patients between 1986 and 2023 are shown in Figure 2. The proportion of male reimbursees started to increase after 1998, growing from 31% to 40% by 2023 (Table 1). Detailed numbers of new, current and deceased reimbursees for each year are reported in Table S1.

**TABLE 1:** Characteristics of glaucoma reimbursee population in 1986–2023.

	1986	1990	1994	1998	2003	2007	2011	2015	2019	2023	1986–2023 change %
Total no. of reimbursees at the end of the year	34 500	42 463	50 896	57 365	66 522	74 088	81 928	89 595	95 554	97 001	181%
New reimbursees	3829	4333	4399	4234	4895	5383	5731	6248	5782	5409	41%
Deceased reimbursees	1685	2272	2708	3073	3377	3497	3845	4114	4472	5154	206%
Net change (new minus deceased reimbursees)	2144	2061	1691	1161	1518	1886	1886	2134	1310	255	−88%
Male/female %	31/69	31/69	31/69	31/69	32/68	34/66	35/ 65	37/63	39/61	40/60	
Number of females	23 691	29 320	35 131	39 416	45 077	49 216	53 288	56 491	58 733	58 395	146%
Number of males	10 809	13 143	15 765	17 949	21 445	24 872	28 640	33 104	36 821	38 606	257%
Finnish population, million	4.9	5.0	5.1	5.2	5.2	5.3	5.3	5.4	5.5	5.6	14%
≥50 year %	28	29	30	33	36	38	39	41	42	42	47%
≥70 year %	9.2	9.5	10	11	12	13	14	15	17	18	100%
Total no. of reimbursees/1000 inhabitants	7.0	8.5	10	11	13	14	15	16	17	17	147%
Females	9.3	11	13	15	17	18	19	20	21	21	121%
Males	4.5	5.4	6.4	7.1	8.4	9.6	11	12	13	14	207%
All reimbursees ≥50 year	23	28	32	32	34	35	37	39	40	40	73%
All reimbursees ≥70 year	50	61	70	73	78	81	82	83	79	76	50%
New reimbursees/1000 inhabitants	0.78	0.87	0.86	0.82	0.94	1.0	1.1	1.1	1.0	0.97	24%
Females	1.0	1.2	1.1	1.1	1.2	1.2	1.3	1.3	1.2	1.0	2%
Males	0.52	0.57	0.57	0.55	0.69	0.77	0.86	0.99	0.93	0.88	70%
New reimbursees ≥50 year	2.5	2.8	2.7	2.3	2.4	2.5	2.5	2.6	2.4	2.2	−12%
New reimbursees ≥70 year	4.9	5.4	5.1	4.2	4.4	4.5	4.5	4.5	3.9	3.6	−27%

**FIGURE 3:**
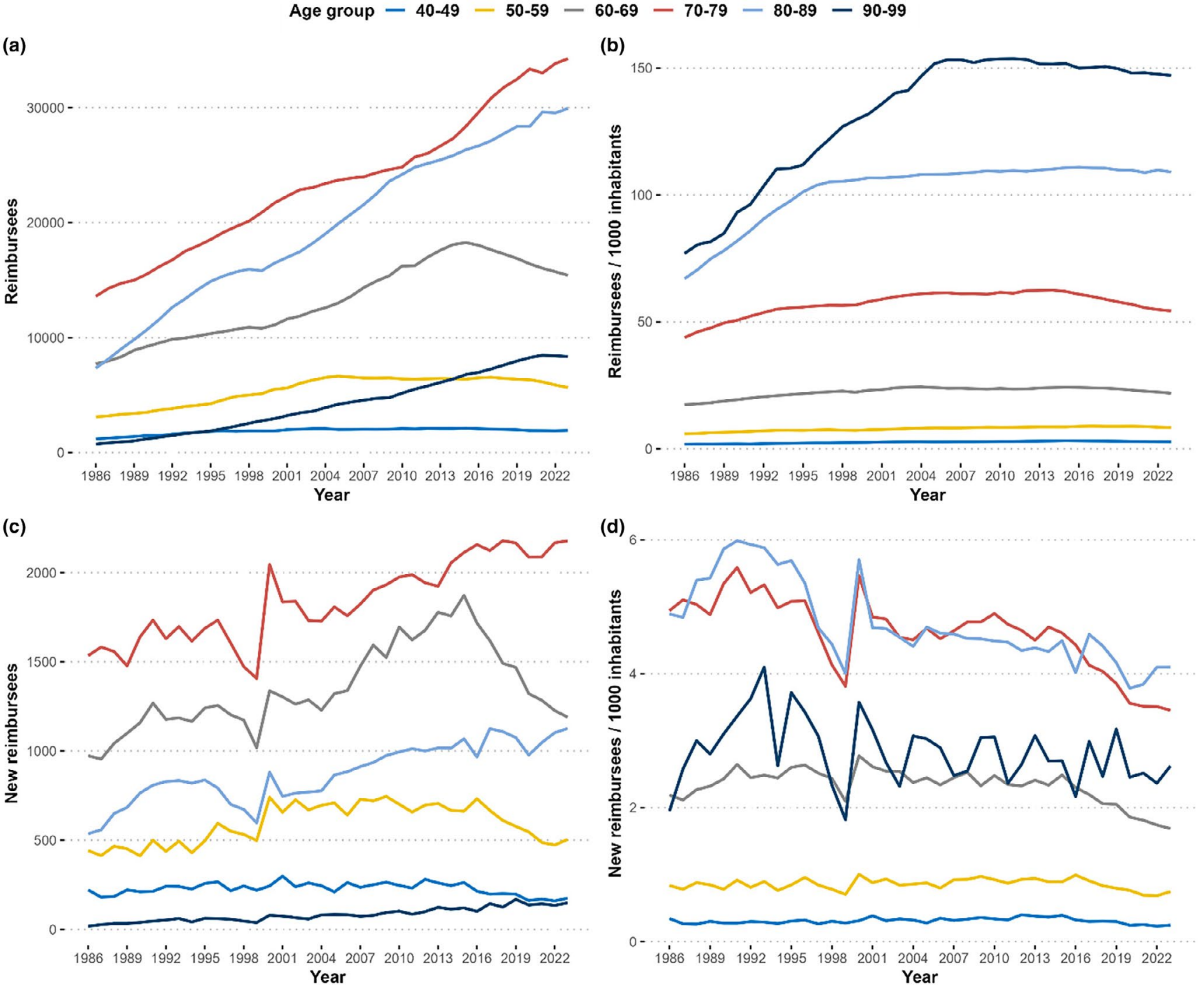
Annual number of absolute (a) and relative (b) number of total glaucoma reimbursees and absolute (c) and relative (d) number of new reimbursees per 10‐year age groups. Lines not drawn for ages below 40 or over 100 years due to low numbers.

The proportion of all glaucoma reimbursees per total Finnish population increased 2.5‐fold over 37 years, from 0.74% in 1986 to 1.8% in 2023 (Table 1). The increase was 1.7‐fold for those over 50‐year‐old (2.3% in 1986 and 4.0% in 2023) and more pronounced among males. In the 10‐year age groups, the largest increases were seen in 1984–2006. After this, the number of reimbursees adjusted for population has stabilized and later started to decrease in all the age groups (Figure 3b).

The number of new reimbursees per year increased 24% (3829 in 1986 and 5409 in 2023) corresponding to an increase from 0.78 to 0.97 new users per 1000 inhabitants, respectively. The number of new reimbursees peaked in 2015 (1.14 per 1000 inhabitants) and after adjusting for age and population, the rate of new reimbursees has been stable or declining since 1990s in all the age groups (Table 1, Figure 3d).

**FIGURE 4:**
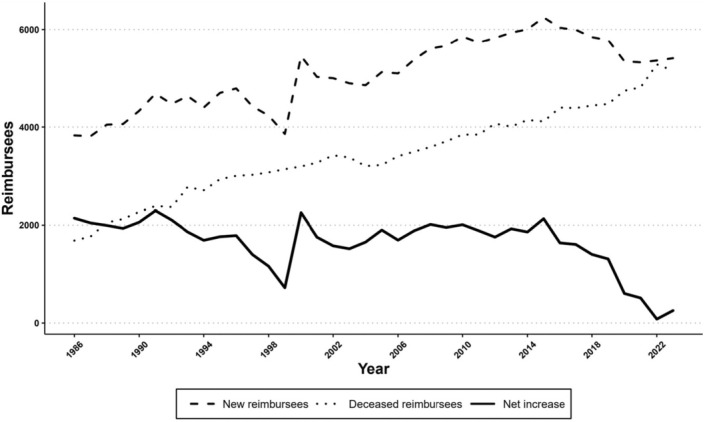
Annual number of new and deceased glaucoma reimbursees and their difference, the net increase.

**FIGURE 5:**
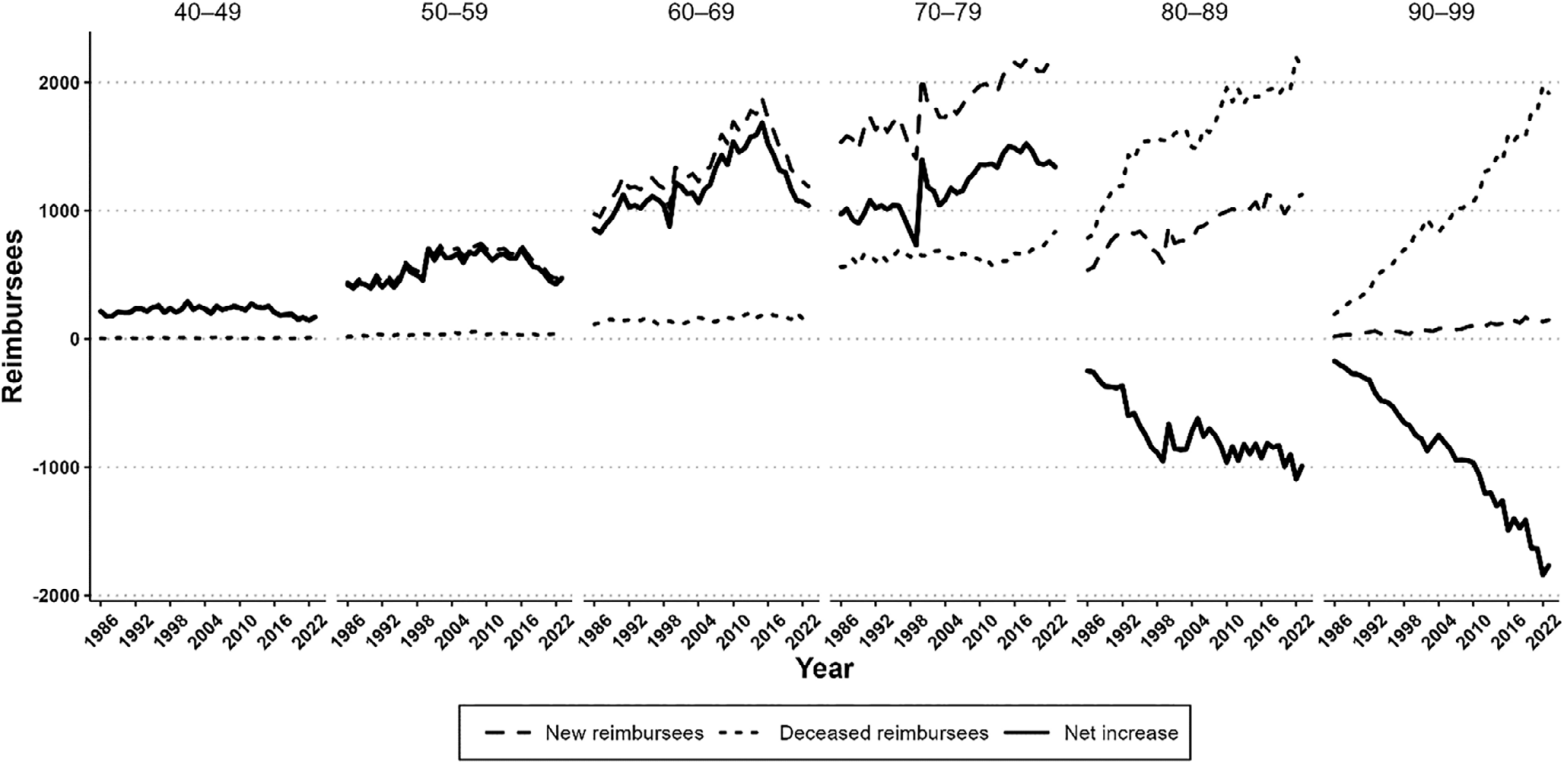
Annual number of new and deceased glaucoma reimbursees and their difference (net increase) by age group. Lines not drawn for ages below 40 or over 100 years due to low numbers.

The net increase in the number of reimbursees (new minus deceased reimbursees) showed a stable upward trend in 1986–2015, excluding the outlier year 1999 which according to Kela was due to a change in its reimbursement policies of certain glaucoma medications (Figure 4). Since 2015, the net increase has decelerated due to decrease in the number of new reimbursees and increase in mortality rate of the glaucoma reimbursee population (Figure S4). For example, in 2023 the net increase of new reimbursees was only 255 patients and 80 patients in 2022. The graphs of net increases in each age group are shown in Figure 5.

The proportion of rejected reimbursement applications has increased since 1992 with an average of 2.7% being rejected. The rejection rate has varied between 0.1% and 5.8%, peaking in 2014, slowing down in 2015–19 and peaking again in 2020–23 to the level of 2014 (Table S5).

## 4 DISCUSSION

The 37‐year increase in the overall number of glaucoma medication users reflects the aging of the population in Finland (Table 1). The increase has also likely been influenced by the National Access to Care Criteria, defined for the first time in 2004, which enabled glaucoma patients receiving public eye care throughout the country (Tuulonen et al., 2009). This improvement may have influenced for example, males' increasing proportion in the new glaucoma medication users (Table 1). Prior to 2004, in southern parts of the country stable glaucoma patients were primarily instructed to use out‐of‐pocket services in private practice (Tuulonen et al., 2009). The Finnish National Criteria for Access to Care are decreed by the Ministry of Social Affairs and Health for all specialities, with the latest update in 2019. All criteria are based on the current care guidelines and defined by all chief physicians in the public sector.

The decelerating trend in 2015–23 in the net change of glaucoma medication users in our study was unexpected, that is, the number of new reimbursees started to approach the number of deceased reimbursees per year (Figure 4). This decline could potentially relate to various factors. Considering firstly Kela's role, the main principles of the reimbursement criteria over time have complied with the Current Care Guidelines (Figure 1). Regarding the reimbursement applications, a study evaluating a sample of them in 1994–2006 reported that while 80% of the applications met all Kela's criteria, all applicants were granted reimbursements (Hagman, 2013). From Kela's data on file, the rejection rates of applications for reimbursing glaucoma medications have generally been low, suggesting that rejections do not represent a major role on the overall trend of medication users (Table S5).

Secondly, changing glaucoma treatment practices over time may also affect the number of medication users. However, considering for example laser trabeculoplasty, it has been used as primary therapy in Finland for almost 40 years (Tuulonen et al., 1985). However, national data on laser procedures as primary or secondary treatments are currently unavailable, and Finland also lacks reliable national statistics on glaucoma surgeries. However, surgical interventions are not generally recommended as primary glaucoma therapy for example in the Finnish and the European Glaucoma Society guidelines (Abegão Pinto et al., 2023; European Glaucoma Society, 2021; Leinonen et al., 2024).

Thirdly, the COVID‐19 pandemic led to increased mortality among elderly Finnish population in 2020–22, thus potentially also contributing to the decline in the number of glaucoma reimbursees in the country. However, the pandemic cannot explain the preceding downward trend seen in 2015–19. Compared to reimbursed medications for systemic diseases in Kela's Info Tray in 2020–22, very few (asthma and epilepsy) showed a declining trend in reimbursees during the pandemic.

Fourthly, the rapidly increasing demand for all eye care services versus limited resources may also affect access to glaucoma care (Tuulonen et al., 2022). For example, while the number of glaucoma visits in Tays Eye Centre increased 30% in 2015–2023, the number of injections for age‐related macular degeneration more than doubled during the same period without corresponding increase in overall resources (data on file).

**FIGURE 6:**
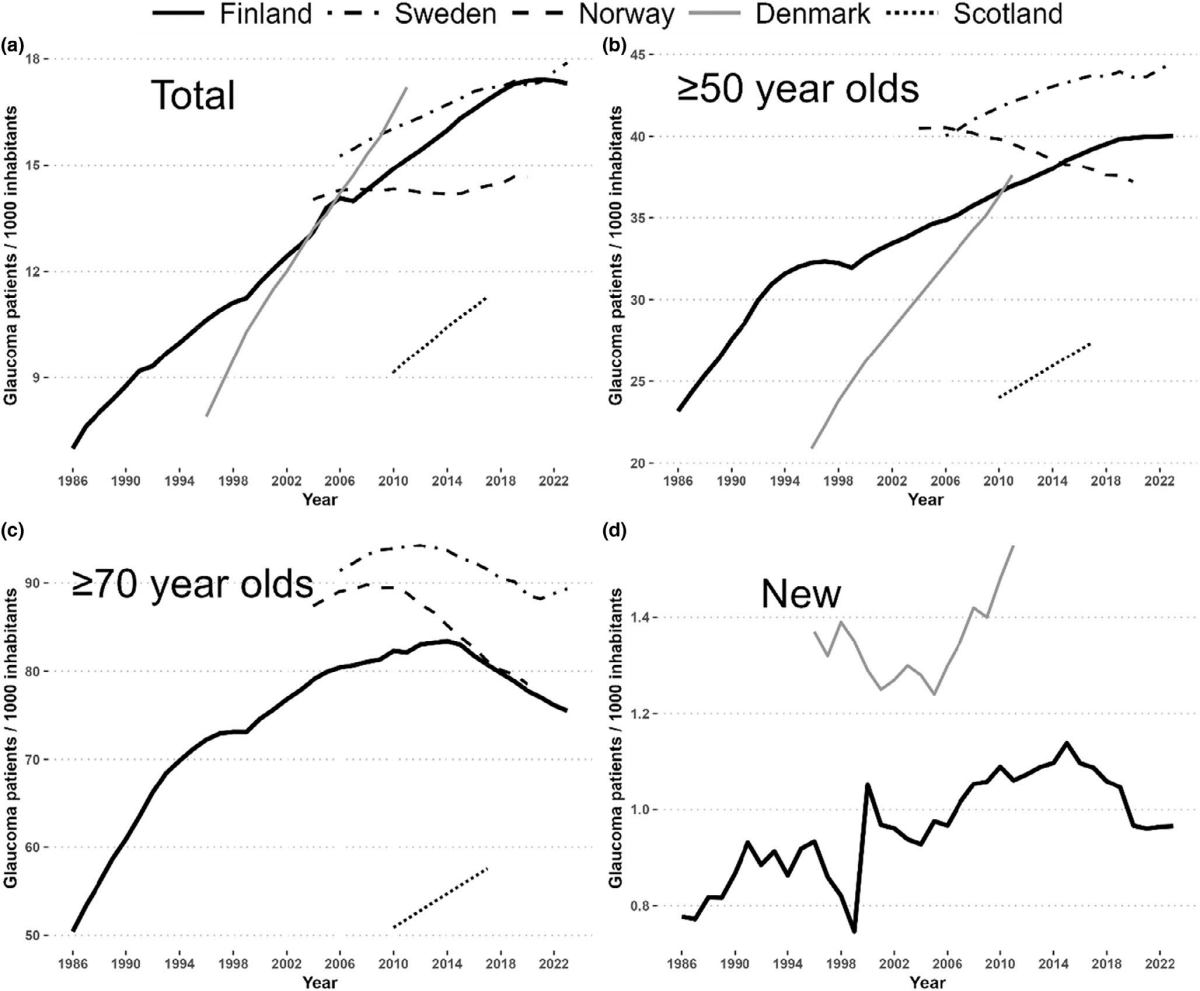
Nordic comparisons of glaucoma drug reimbursees (Finland) and prescribees (Denmark, Norway, Scotland and Sweden) with total numbers (a), over 50‐year‐olds (b), over 70‐year‐olds (c) and new reimbursees (d). Sources for numbers outside Finland are Kolko et al. (2015, Denmark), Rotchford et al. (2020, Scotland), Socialstyrelsen (Sweden), Folkhelseinstituttet and Slettedal et al. (Norway). Data is also available in Table S2.

Compared to published studies in other countries, a 14‐year study in Norway showed only a minimal increase in glaucoma medication users, with a decline among older patients (Slettedal et al., 2020; Figure 6). Denmark also reported steady rises in the number of glaucoma patients until 2018, followed by a stable trend (Horwitz et al., 2023; Kolko et al., 2015). Finland's rate of new users per 1000 people was about 30% lower than in Denmark and Norway during comparable periods (Slettedal et al., 2020; Table S2).

Outside Nordic countries, the population‐adjusted numbers of glaucoma medication users in Scotland were lower compared to Finland (Rotchford et al., 2020) (Figure 6). Japan has forecast a decline in glaucoma patients starting in 2025 (Sakamoto et al., 2017). In England and Australia, the numbers of glaucoma prescriptions have increased during the 2000th century and peaked in the 2010s with subsequent decrease (Connor & Fraser, 2014; Newman & Andrew, 2019). Connor and Fraser (2014) reported that the introduction of NICE guidelines (National Institute of Health for Care and Excellence, 2017) did not change glaucoma prescribing practices. Interestingly, similar to the current study, Perera et al. (2020) reported in Australia a downward trend in the number of medications prescribed in 2015–17.

A key strength of this study is the availability of comprehensive national data on glaucoma drug reimbursements since 1986. Kela's reimbursement database covers 97%–99% of glaucoma patients in Finland and includes also the number of patients not reclaiming medications from pharmacies. In contrast, the Danish data indicated that up to 10% of glaucoma patients may not reclaim medications despite their prescriptions (Horwitz et al., 2023). The obvious limitation of this study relates to the fact that reimbursement data does not reflect ‘true’ number of glaucoma patients. For instance, patients with ocular hypertension with IOP below the 30 mmHg may be given a glaucoma diagnosis to gain reimbursement eligibility, potentially leading to overdiagnosis (Tuulonen et al., 2009). The compliance of ophthalmologists to comply with the ‘2 out of 3’‐recommendation for diagnosis is not known either. As reimbursement rights are granted for life, historical overtreatment may still influence the current trends in the data. Moreover, we lack statistics on patients primarily treated with laser trabeculoplasty, who may not require medication.

## 5 CONCLUSIONS

This 37‐year study of glaucoma medication reimbursements in Finland revealed a steady increase in the overall number of reimbursees, reflective of the aging population and improvement in access to eye care. However, from 2015 to 2023, we observed an unexpected deceleration in the net increase of glaucoma medication users. This trend diverges from previous forecasts for both Finnish and global trends which anticipate a continuous increase in the number of glaucoma patients. Whether the downward trend in the net users of glaucoma medications represents a transient, or a continuous phenomenon in Finland calls for further follow‐up as well as updating our previous prediction model (Tuulonen et al., 2009) using the history data of this study up to 2023.

## AUTHOR CONTRIBUTIONS

All authors fill all four criteria for authorship by International Committee of Medical Journal Editors (ICMJE).

## FUNDING INFORMATION

The study was supported by the State funding for university‐level health research, Tampere University Hospital, Wellbeing Services County of Pirkanmaa (T63464, T64484), Tampere University Hospital Support Foundation, Tampere University Hospital, Wellbeing Services county of Pirkanmaa (T64124), LUX—Foundation for Glaucoma Research for HUJ.

## CONFLICT OF INTEREST STATEMENT

EL, AT and KL: None to declare. SL: Lecture fees from Santen, Thea and Abbvie. HUJ: Advisory board member of Abbvie, Bayer, Novartis and Roche, lecture fees from Santen, Thea and Roche.

## Supporting information


Data S1

## Data Availability

The links to the publicly available data sets used in the study are provided. Additionally, the study data is provided in Tables S2 and S3.
