# Supplementary material for: Users of reimbursed glaucoma medications in Finland in 1986–2023: A nationwide study
Source: Acta Ophthalmol. 2024 Dec 9;103(3):348–56. doi: 10.1111/aos.16803 (PMC11986400; doi:10.1111/aos.16803)
Supplement: Supplementary file 1 — Data S1 [file AOS-103-348-s001.zip › supporting_information2.docx]

**Supplementary Figure S4.** Mortality rate of reimbursees by age group

**Supplementary Table S5.** Annual number of total and rejected glaucoma reimbursement applications

This supplemental material has been provided by the authors to give readers additional information about their work.

**Figure S4. Mortality rate of reimbursees by age group**


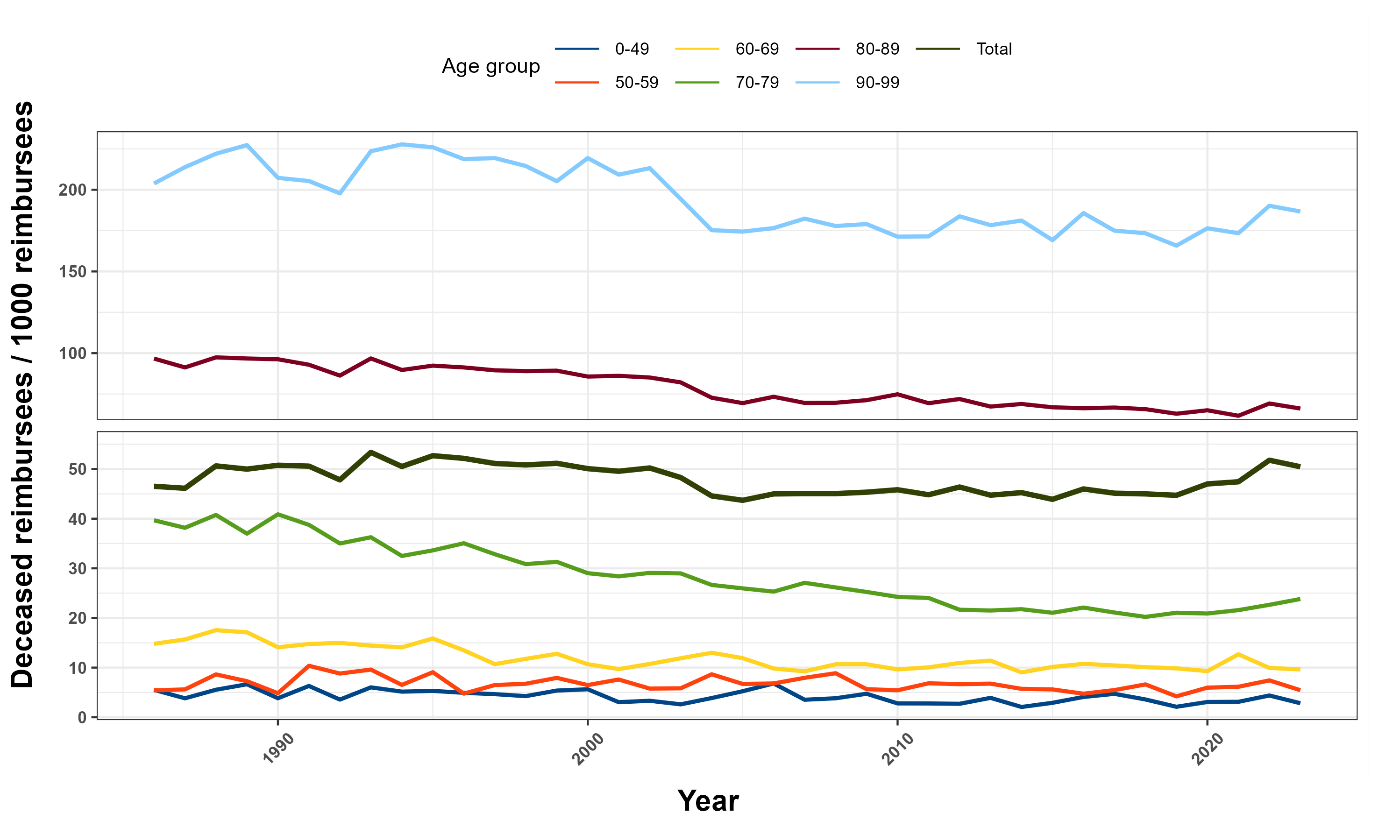


**Table S5.** **Annual number of total and rejected glaucoma reimbursement applications**

| Year of decision | Total reimbursement applications | Rejected applications | New reimbursees | Rejection rate (%) |
| --- | --- | --- | --- | --- |
| 1992 | 4817 | 3 | 4472 | 0.06 |
| 1993 | 4852 | 9 | 4635 | 0.19 |
| 1994 | 4488 | 54 | 4399 | 1.20 |
| 1995 | 4809 | 51 | 4698 | 1.06 |
| 1996 | 5140 | 49 | 4790 | 0.95 |
| 1997 | 4658 | 37 | 4423 | 0.79 |
| 1998 | 4536 | 49 | 4234 | 1.08 |
| 1999 | 4073 | 35 | 3859 | 0.86 |
| 2000 | 5718 | 71 | 5452 | 1.24 |
| 2001 | 5615 | 77 | 5026 | 1.37 |
| 2002 | 5500 | 72 | 5000 | 1.31 |
| 2003 | 5472 | 101 | 4895 | 1.85 |
| 2004 | 5581 | 106 | 4856 | 1.90 |
| 2005 | 5391 | 117 | 5125 | 2.17 |
| 2006 | 5347 | 133 | 5098 | 2.49 |
| 2007 | 5497 | 128 | 5383 | 2.33 |
| 2008 | 5890 | 149 | 5609 | 2.53 |
| 2009 | 5915 | 151 | 5662 | 2.55 |
| 2010 | 6073 | 144 | 5856 | 2.37 |
| 2011 | 5880 | 142 | 5731 | 2.41 |
| 2012 | 6050 | 202 | 5826 | 3.34 |
| 2013 | 6268 | 275 | 5935 | 4.39 |
| 2014 | 6405 | 319 | 6005 | 4.98 |
| 2015 | 6510 | 291 | 6248 | 4.47 |
| 2016 | 6214 | 214 | 6036 | 3.44 |
| 2017 | 6227 | 206 | 5995 | 3.31 |
| 2018 | 5933 | 169 | 5843 | 2.85 |
| 2019 | 6149 | 193 | 5782 | 3.14 |
| 2020 | 5515 | 316 | 5346 | 5.73 |
| 2021 | 5639 | 325 | 5325 | 5.76 |
| 2022 | 5630 | 292 | 5362 | 5.19 |
| 2023 | 5918 | 270 | 5409 | 4.56 |

The data is only available starting from 1992. Note that number of applications does not necessarily match the number of new reimbursees in a given year due to the possibility to submit several applications and timing differences.
